# IL-8和MMP-9在非小细胞肺癌患者组织及血清中表达水平的研究

**DOI:** 10.3779/j.issn.1009-3419.2010.08.09

**Published:** 2010-08-20

**Authors:** 志东 刘, 绍发 许, 宁 肖, 长兴 宋, 海青 张, 福根 李

**Affiliations:** 1 101149 北京，北京市结核病胸部肿瘤研究所胸外科 Department of Thoracic Surgery, Beijing Tuberculosis and Thoracic Tumor Research Institute, Beijing 101149, China; 2 101149 北京，北京市结核病胸部肿瘤研究所临床免疫科 Department of Clinical Immunity, Beijing Tuberculosis and Thoracic Tumor Research Institute, Beijing 101149, China; 3 101149 北京，北京市结核病胸部肿瘤研究所病理科 Department of Pathology, Beijing Tuberculosis and Thoracic Tumor Research Institute, Beijing 101149, China

**Keywords:** 肺肿瘤, 白细胞介素-8, 基质金属蛋白酶-9, 免疫组织化学, 酶联免疫吸附实验, Lung neoplasms, Interleukin-8, Matrix metalloproteinase-9, Immunohistochemistry, Enzyme-linked immunosorbent assay

## Abstract

**背景与目的:**

白细胞介素-8（interleukin-8, IL-8）曾被认为是一种具有趋化作用的炎症因子，近年的研究认为它和基质金属蛋白酶-9（matrix metalloproteinase-9, MMP-9）均为与肿瘤生长和转移密切相关的细胞因子，本研究通过检测IL-8、MMP-9在非小细胞肺癌患者组织和血清中的表达，研究二者与临床病理特征之间的联系，分析肺癌组织和血清中IL-8、MMP-9表达的相关性，并探讨IL-8、MMP-9作为肿瘤标志物评估非小细胞肺癌患者病情进展程度的效能。

**方法:**

应用酶联免疫吸附实验（enzyme-linked immunosorbent assay, ELISA）检测141例非小细胞肺癌患者、40例健康人和40例肺良性疾病患者血清中IL-8、MMP-9的水平。采用免疫组化SP法检测95例非小细胞肺癌、21例良性疾病肺组织及25例正常肺组织中IL-8和MMP-9的表达。

**结果:**

IL-8、MMP-9在非小细胞肺癌患者血清和组织的表达水平明显高于肺良性疾病对照组和健康对照组，其差异有统计学意义，且随着临床病理分期的升高而升高。在肺癌患者组织和血清中，IL-8与淋巴转移均有较强的相关性，在肺癌组织中IL-8和MMP-9的表达具有很强的相关性（*r*=0.765）。

**结论:**

IL-8、MMP-9表达水平与非小细胞肺癌的进展密切相关，特别是IL-8与肺癌的淋巴转移具有明显的相关性，其机制可能是通过上调MMP-9实现的。

## Abstract (en)

**Background and objective:**

IL-8 (interleukin-8) has been identified as a chemotactic factor, but recent found that IL-8 and matrix metalloproteinase-9 (MMP-9) are important cytokines which are closely related to the growth and metastasis of tumor. The aim of this study is to explore the relationship between IL-8, MMP-9 expressions and clinical pathological features of non-small cell lung cancer (NSCLC) patients and evaluate the diagnostic potential of IL-8, MMP-9 as tumor markers.

**Methods:**

The serum levels of IL-8 and MMP-9 were detected by enzyme-linked immunosorbentassay (ELISA) in 141 NSCLC patients, 40 healthy adults and 40 patients with benign pulmonary disease. The expressions of IL-8 and MMP-9 were detected by immunohistochemical method in 95 NSCLC tissues, and 21 benign disease lung tissues, 25 normal lung tissues as control.

**Results:**

The level of expression of IL-8 and MMP-9 in serum and tissue of NSCLC was significantly higher than that of healthy and benign respiratory disease, and the expression was gradually increased with the upgrade of clinicopathological stage. The serum and tissue expression of IL-8 and MMP-9 in NSCLC patients with lymph node metastasis was remarkably higher than that without lymph node metastasis. There is an positive correlation (*r*=0.765) between IL-8 and MMP-9 in the tissue of NSCLC patients.

**Conclusion:**

This study has confirmed that IL-8, MMP-9 expressions are related to the development of NSCLC. There is an obvious correlation between IL-8 expression and lymph node metastasis, IL-8 may facilitate the lymph node metastasis by up-regulating MMP-9 expression. Serum level of IL-8 is a valuable auxiliary parameter in diagnosing lymph node metastases of NSCLC with good sensitivity and specificity.

肺癌是目前肿瘤致死的第一位病因，80%以上的肺癌患者被确诊时已属中晚期，5年生存率仅14%^[1]^。侵袭和转移是造成肺癌临床治疗失败和患者死亡的主要原因^[2]^。随着分子生物学技术的发展，人们在不断地寻找、筛选与肺癌侵袭和转移相关的肿瘤标记物，以更好地判断肺癌患者的进展情况，并期待能将其应用于治疗，提高治疗效果。

白细胞介素-8（interleukin-8, IL-8）是Yoshimura等^[3]^于1987年首次从脂多糖植物血凝素刺激人血单核细胞培养上清液中发现的一种趋化因子，以前人们发现它有激活和趋化嗜中性粒细胞的作用，对炎症和免疫过程有着重要的调节作用，近年来有文献报道肿瘤细胞也可分泌IL-8，并发现IL-8可以促进新生血管形成^[4]^、上调基质金属蛋白酶的表达^[5]^，与恶性肿瘤的生长、侵袭和转移密切相关。

细胞外基质和基底膜是阻止肿瘤侵袭和转移的重要组织屏障，基质金属蛋白酶（matrix metalloproteinase, MMP）是降解细胞外基质最重要的酶类，在肿瘤的侵袭和转移中起着重要的作用。基质金属蛋白酶- 9（MMP-9）是基质金属蛋白酶中的一员，分子量为95 kDa，能降解破坏ECM中最主要的组分Ⅳ、Ⅴ型胶原和明胶^[6]^来增强肿瘤的侵袭性和转移能力。

本研究运用酶联免疫吸附实验、免疫组织化学两种方法，分别检测治疗前非小细胞肺癌患者的血清及肺癌组织IL-8、MMP-9的表达水平，分析IL-8、MMP-9的表达与肺癌患者临床病理因素之间的联系，分析IL-8、MMP-9表达的相关性，探讨IL-8参与非小细胞肺癌转移的机制。

## 1 材料与方法

### 1.1 病例资料

① 血清组：非小细胞肺癌患者：141例，男性107例，女性34例，年龄31岁-82岁，中位年龄61岁，为连续选取2007年1月-2007年11月到北京胸科医院胸外科及呼吸科就诊的患者。患者均经细胞学或组织学病理证实为非小细胞肺癌，未经手术、放疗、化疗及生物靶向治疗等特异抗肿瘤治疗，无心脑血管并发症，近期无急性感染。患者经血清IL-8、MMP-9检测后行手术治疗者95例，进行其它治疗者46例。非小细胞肺癌患者的临床病理分期及远处转移的判断遵循国际抗癌协会（International Union Against Cancer, UICC）1997年公布的肺癌TNM分期。良性疾病对照组：40例，男性25例，女性15例，年龄15岁-80岁，中位年龄52岁，为同期连续选取在北京胸科医院胸外科及呼吸科就诊的患者，病理类型包括肺结核8例、急性肺炎8例、阻塞性肺疾病4例、支气管扩张12例、肺良性肿块8例。健康对照组：40例，男性30例，女性10例，年龄25岁-68岁，中位年龄56岁，均为无心、肝、肺和肾等重要器官疾病，肝肾功能正常者。

② 免疫组化组：非小细胞肺癌：为血清组非小细胞肺癌患者中行手术治疗的患者，共95例，男性79例，女性16例，年龄31岁-79岁，中位年龄60岁。良性疾病：为血清组良性疾病对照患者行手术治疗者21例。正常肺组织：距癌组织 > 5 cm，经HE染色证实无癌细胞浸润的肺组织视为正常肺组织，本实验取25例。

### 1.2 酶联免疫吸附实验

患者和健康人均在清晨空腹抽取外周静脉血5 mL，取血后当日离心（1 000 rpm）10 min，分离血清，置于-40 ℃保存待测定。鼠抗人IL-8、MMP-9单克隆抗体购自美国R & D公司，经晶美生物工程有限公司包埋成ELISA试剂盒，血清IL-8、MMP-9水平测定严格按ELISA试剂盒说明书进行，由专用电脑测量出具体数值。所有病例均双标本测定，取中间值。

### 1.3 免疫组化（SP法）

#### 1.3.1 免疫组化方法

所有标本均用10%中性甲醛溶液固定，石蜡包埋，制成厚5 μm的切片，采用免疫组化SP法进行IL-8、MMP-9染色。鼠抗人IL-8、MMP-9单克隆抗体购自美国R & D公司，SP试剂盒、DAB显色剂购自泉晖生物技术有限公司。免疫组化染色按SP试剂盒操作说明进行，以高压加热修复法进行抗原修复，4 ℃过夜，采用PBS代替一抗做阴性对照，阳性对照采用已知实验中证实对本实验所用抗体呈阳性染色反应的非小细胞肺癌组织阳性切片。

#### 1.3.2 结果判定

每个切片随机观察5个高倍镜视野，每个高倍镜视野计数200个肿瘤细胞，分别由两位病理科医生对染色结果进行评判，IL-8、MMP-9阳性着色定位于细胞浆或细胞膜，细胞被染色为棕黄色或棕褐色为阳性，按着色强度计分：无着色为0分; 棕黄色清晰为2分; 着色强度介于二者之间为1分，棕褐色为3分。按着色细胞百分数计分：着色细胞 < 30%为0分; ≥30%但 < 60%为1分，≥60%为2分。将着色细胞着色强度计分与着色百分数计分相加，总计0分或1分为（-），总计2分或3分为（+），总计4分为（++），总计5分为（+++）。

### 1.4 统计学处理

血清IL-8、MMP-9水平所测数据采用SPSS 13.0软件包进行分析，经正态性检验，IL-8、MMP-9血清浓度数据成偏态分布，多组间数据差异比较应用*Kruskal-Wallis H*检验，两组间差异比较用*Mann-Whitney* U检验。免疫组化IL-8、MMP-9的表达与临床病理特征关系的比较，采用四格表或行×列表χ^2^检验。组间相关性采用*Spearman*相关分析。以*P* < 0.05为差异具有统计学意义。诊断效能的评价采用ROC曲线计算灵敏性和特异性，并计算*Youden’s*指数、阳性预测值、阴性预测值。

## 2 结果

### 2.1 非小细胞肺癌患者血清IL-8、MMP-9表达水平与临床病理特征的关系

如表 1所示，非小细胞肺癌患者（包括行手术治疗和未行手术治疗的共141例患者）血清IL-8、MMP-9水平明显高于肺良性疾病对照组及健康对照组，其差异有统计学意义。非小细胞肺癌患者血清IL-8、MMP-9表达水平随着临床TNM病理分期的升高而升高，其差异有统计学意义。在经手术治疗的95例非小细胞肺癌患者中，血清IL-8、MMP-9的表达水平与肿瘤的淋巴转移和远处转移有关，有淋巴转移或远处转移的表达水平高于无转移的患者，其差异有统计学意义。MMP-9的表达水平与肿瘤的T分期相关，但血清IL-8表达水平与肿瘤T分期相关性却无明显的统计学意义（*P*=0.080）。血清IL-8、MMP-9的表达水平与肿瘤大小无明显的统计学意义（*P*=0.071）。从表 1还可看出，非小细胞肺癌患者的血清IL-8、MMP-9水平与肺癌的病理类型（本实验含鳞癌40例，腺癌50例，其它病理类型包括腺鳞癌、大细胞癌共5例，由于数据相差悬殊，经检验其结果无统计学意义，而鳞癌和腺癌的比较结果有统计学意义，故在表中仅列出了鳞癌和腺癌的例数）、分化程度、肿瘤位置无关，且与患者的性别、年龄、吸烟史也无关。

**1:** 非小细胞肺癌患者血清IL-8、MMP-9表达水平与临床病理特征的关系 The relationship between serum IL-8 and MMP-9 and the clinicopathological characteristics

Variables	*n*	IL-8 (pg/mL)		*P*	MMP-9 (ng/mL)		*P*
		Median	Quartile		Median	Quartile	
Group				< 0.001			< 0.001
Health	40	16.55	11.83-35.18		29.41	18.81-56.11	
Benign disease	40	27.20	13.43-541.35		51.98	20.29-87.52	
Non-small cell lung cancer	141	138.10	42.55-327.95		88.49	39.19-145.64	
Pathologic stage				< 0.001			< 0.001
Stage Ⅰ	38	33.10	19.75-78.10		41.86	22.95-113.83	
Stage Ⅱ	21	110.60	21.15-418.10		27.16	19.19-131.46	
Stage Ⅲ	53	163.00	102.05-322.80		89.51	59.10-122.96	
Stage Ⅳ	29	331.30	198.90-581.20		149.70	109.07-240.07	
Tumor status				0.080			0.006
T1	17	55.60	21.95-223.15		40.63	18.75-103.98	
T2	54	89.55	31.38-227.80		60.65	28.88-120.42	
T3	17	110.60	19.05-274.10		68.74	23.71-112.29	
T4	7	370.70	157.00-662.20		240.06	103.74-351.37	
Lymph node status				< 0.001			0.002
N0	51	33.10	17.90-91.30		40.63	19.02-113.55	
N1	12	328.75	84.75-647.13		149.68	39.60-211.77	
N2	32	152.05	111.85-315.40		88.36	58.04-115.39	
Distant metastasis				0.006			0.001
Present	89	91.30	23.20-222.60		59.28	25.00-116.58	
Absent	6	395.95	303.35-486.30		240.07	129.84-316.95	
Tumor size				0.071			0.071
≤3 cm	34	56.45	19.75-165.20		40.10	19.63-106.19	
> 3 cm	61	114.90	34.55-320.55		77.13	41.81-128.45	
Histology				0.229			0.685
Adenocarcinoma	50	98.20	22.08-226.43		65.81	26.94-116.54	
Squamous cell carcinoma	40	97.70	34.08-571.50		60.71	26.29-130.06	
Differentiation				0.611			0.395
Poorly	9	94.70	24.60-106.55		56.98	21.77-85.97	
Moderately	76	110.90	27.48-308.73		71.35	25.54-135.03	
Well	10	99.95	21.93-850.00		44.14	27.37-132.35	
Location				0.672			0.757
Central	37	96.10	22.60-267.10		75.19	29.96-116.58	
Peripheral	58	105.25	31.23-314.20		59.10	23.38-136.74	
Gender				0.929			0.164
Male	79	110.60	23.00-304.30		68.74	37.50-119.83	
Female	16	88.65	27.98-287.40		27.59	15.52-141.22	
Age (years)				0.926			0.465
≤60	44	93.00	22.20-308.73		55.43	21.88-118.26	
> 60	51	105.10	26.20-226.20		77.13	30.17-136.14	
Smoking				0.154			0.399
Non-smoker	38	82.55	22.60-219.13		55.04	20.80-124.57	
Smoker	57	114.90	25.90-328.75		73.95	33.62-122.53	

### 2.2 肺癌组织中IL-8、MMP-9免疫组化表达水平与临床病理特征的关系

免疫组化结果如图 1和图 2所示，表 2（本表结果为计量资料，将TNM分期、肿瘤大小及淋巴结分开统计后，经统计检验发现有些亚组数值太小，达不到统计要求，故不得不将TNM分期的Ⅲ期、Ⅳ期合并，T3和T4合并，淋巴结分组中N1、N2、N3合并）所示，非小细胞肺癌组织的IL-8、MMP-9水平明显高于良性疾病肺组织及健康肺组织，其差异有统计学意义。非小细胞肺癌组织IL-8、MMP-9的表达水平随着临床TNM病理分期的升高而升高，其差异有统计学意义。非小细胞肺癌组织IL-8、MMP-9的表达水平与肿瘤的淋巴转移有关，有淋巴转移的表达水平高于无转移的肺癌组织，其差异有统计学意义。但二者与远处转移的相关性却无显著的统计学意义。从表 2还可以看出，非小细胞肺癌组织的IL-8、MMP-9水平与肺癌的肿瘤大小、病理类型（仅包括鳞癌和腺癌）、分化程度、肿瘤位置无关，且与患者的性别、年龄、吸烟史也无关。

**1:**
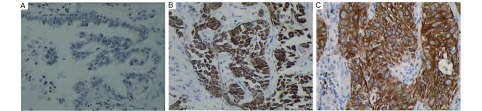
IL-8在肺腺癌和鳞癌组织中表达的免疫组化染色。IL-8蛋白表达于肺癌细胞胞浆和胞膜，苏木素染色（SP, ×400）。A：阴性对照; B：腺癌染色阳性; C：鳞癌染色阳性。 IL-8 expression in lung adenocarcinoma and squamous carcinoma. Immunohistochemical staining of IL-8 protein was located in cytoplasm and cell membrane (SP, ×400). A: negative control; B: adenocarcinoma positive; C: squamous positive.

**2:**
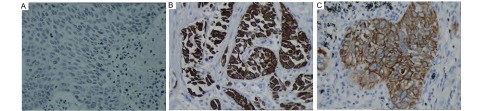
MMP-9在肺腺癌和鳞癌组织中表达的免疫组化染色。MMP-9蛋白表达于肺癌细胞胞浆和胞膜，苏木素染色（SP, ×400）。A：阴性对照; B：腺癌染色阳性; C：鳞癌染色阳性。 MMP-9 expression in lung adenocarcinoma and squamous carcinoma. Immunohistochemical staining of MMP-9 protein was located in cytoplasm and cell membrane (SP, ×400). A: negative control; B: adenocarcinoma positive; C: squamous positive.

**2:** 肺癌组织中IL-8、MMP-9免疫组化表达水平与临床病理特征的关系 The relationship between tissue IL-8 and MMP-9 expressions and the clinicopathological characteristics

Variables	*n*	IL-8 status		*P*	MMP-9 status		*P*
		Positive	Negative		Positive	Negative	
Group				< 0.001			< 0.001
Normal lung tissue	25	2	23		3	22	
Benign lung tissue	21	4	17		6	15	
Lung cancer tissue	95	60	35		62	33	
Pathologic stage				0.002			0.022
Stage Ⅰ	38	18	20		20	18	
Stage Ⅱ	19	10	9		11	8	
Stage Ⅲ, Ⅳ	38	32	6		31	7	
Tumor status				0.504			0.071
T1	17	9	8		7	10	
T2	54	24	20		38	16	
T3, T4	24	17	7		17	7	
Lymph node status				0.008			0.022
N0	51	26	25		28	23	
N1, N2, N3	44	34	10		34	10	
Distant metastasis				0.082			0.089
Present	89	54	35		56	33	
Absent	6	6	0		6	0	
Tumor size				0.513			0.325
< 3 cm	34	20	14		20	14	
> 3 cm	61	40	21		42	19	
Histology				0.206			0.730
Adenocarcinoma	50	34	16		33	17	
Squamous cell carcinoma	40	22	18		25	15	
Differentiation				0.721			0.061
Poorly	9	5	4		3	6	
Moderately, Well	86	55	31		59	27	
Location				0.251			0.413
Central	37	26	11		26	11	
Peripheral	58	34	24		36	22	
Gender				0.530			0.748
Male	79	51	28		51	28	
Female	16	9	7		11	5	
Age (years)				0.606			0.064
< 60	44	29	15		33	11	
> 60	51	31	20		29	22	
Smoking				0.385			0.429
Non-smoker	38	22	16		23	15	
Smoker	57	38	19		39	18	

### 2.3 95例手术非小细胞肺癌患者血清及肺组织中IL-8、MMP-9表达水平、肺组织IL-8、MMP-9表达水平与临床病理T、N、M特征的相关性

从表 3可以看出，在各种因素的相关系数分析中，非小细胞肺癌患者组织中IL-8、MMP-9表达的相关性最强（*r*=0.765），其次为血清IL-8的表达与淋巴转移的相关性（*r*=0.515），血清中IL-8、MMP-9表达也具有较强的相关性（*r*=0.445）。

**3:** 95例手术非小细胞肺癌患者血清及肺组织IL-8、MMP-9表达水平与临床病理T、N、M特征的相关性 *Spearman* analysis of the correlation between IL-8, MMP-9 of serum and tissue and T, N, M status of 95 NSCLC patients

	IL-8			MMP-9		T	N	M
	Serum	IHC		Serum	IHC			
IL-8								
Serum	1	0.311^**^		0.445^**^	-	0.148	0.515^**^	0.286^**^
IHC	0.311^**^	1		-	0.765^**^	0.182	0.426^**^	0.241^*^
MMP-9								
Serum	0.445^**^	-		1	0.119	0.222^*^	0.303^**^	0.342^**^
IHC	-	0.765^**^		0.119	1	0.261^*^	0.419^**^	0.216
^**^Correlation is significant at the 0.01 level (2-tailed). ^*^Correlation is significant at the 0.05 level (2-tailed).								

### 2.4 IL-8、MMP-9作为肿瘤标志物对非小细胞肺癌病情进展的评价效能

从表 4可以看出，但IL-8、MMP-9对于预测早期肺癌（尚未出现淋巴转移）的发生效能较差（当特异性为75%左右时，其敏感性仅为21.1%和31.6%，约登指数及阳性预测值、阴性预测值也较低）。IL-8对于预测肺癌的淋巴转移具有较好的敏感性（76.6%）和特异性（79.2%），约登指数及阳性预测值、阴性预测值也较高。

**4:** IL-8、MMP-9作为肿瘤标志物对非小细胞肺癌病情进展的评价效能 Comparison of IL-8, MMP-9 performances as possible indicators of cancer presence and dissemination

Marker category	AUC (95%CI); (AUC=0.5)	*P*	Optimal cut-off	Sensitivity and specificity	*Youden's* index	Likelihood ratios (PV+/PV-)
NSCLC presence						
IL-8	0.588 (0.481-0.695)	0.123	86.35 pg/mL	21.1%, 76.2%	-0.027	0.296/0.670
MMP-9	0.555 (0.439-0.671)	0.333	76.36 ng/mL	31.6%, 77.5%	0.091	0.400/0.705
Cancer disseminaton						
IL-8	0.811 (0.719-0.904)	< 0.001	110.90 pg/mL	76.6%, 79.2%	0.558	0.783/0.776
MMP-9	0.700 (0.594-0.806)	0.001	79.77 pg/mL	63.8%, 72.9%	0.367	0.698/0.673
Distant metastasis						
IL-8	0.763 (0.681-0.846)	< 0.001	313.20 pg/mL	62.1%, 77.7%	0.398	0.419/0.888
MMP-9	0.817 (0.740-0.893)	< 0.001	126.19 pg/mL	69.0%, 79.5%	0.485	0.465/0.908
ROC curve: receiver operating characteristics curve; CI: confidence intervals.						

## 3 讨论

本研究发现IL-8、MMP-9在非小细胞肺癌患者血清及肿瘤组织中的表达水平与肺癌的进展密切相关，二者之间也有密切的相关性。

肿瘤是依赖血管发生的，并且肿瘤的生长需要伴随血液供应的增加，无论是原发部位还是转移病灶，肿瘤的持续生长必须依赖新生血管的形成^[7]^，在局部缺乏毛细血管增殖的条件下，肿瘤的生长不会超出2 mm-3 mm。新生血管不仅提供了肿瘤生长所必须的营养，为转移提供了通路，而且新生血管基底膜呈节段性分布，比正常组织的成熟毛细血管对瘤细胞有更高的亲和性，所以血管新生是影响肿瘤大小、局部和远处转移的重要因素。

1992年Strieter^[8]^在做兔角膜实验中第一次发现IL-8具有促进血管形成的作用。Smith等^[9]^发现，支气管肺癌肿瘤细胞内的IL-8含量较正常组织高4倍。后来Arenberg等^[10]^也发现IL-8参与NSCLC内新生血管的形成。目前已有多篇研究报道在胃癌^[11]^、胰腺癌^[12]^、结直肠癌^[13]^和乳腺癌^[14]^患者的肿瘤组织中IL-8水平表达明显升高，本实验检测肺癌组织中IL-8的表达也得出了类似的结论。本研究显示，从总体上看，非小细胞肺癌患者IL-8的血清水平和在组织中的表达水平均明显高于良性疾病对照组及健康对照组，而且其水平随着肺癌临床TNM病理分期的升高而升高，其差异有统计学意义。但在血清IL-8检测中，无淋巴转移的Ⅰ期肺癌患者与健康对照组及良性疾病对照组却无明显的差异，而且，不同肺癌T分期、不同肿瘤大小组的血清IL-8表达水平差异分别为*P*=0.080和*P*=0.071，虽有不同的倾向但并无显著的统计学差异。这可能来自两个原因：一方面，本实验虽总例数较多，但分散至各组则例数较少，可能是例数不足，不能真正代表全体; 另一方面，血清IL-8水平并不能直接反映组织中IL-8的表达：肺癌组织中局部IL-8水平增高，但只有少量能释放入血，血清中的IL-8可来源于肿瘤细胞，也可来源于中性粒细胞、单核细胞等炎性细胞，血清中炎性细胞分泌IL-8掩盖了肺癌组织分泌IL-8的差异。

在肺癌患者中，血清IL-8水平与淋巴结转移、远处转移相关，而且血清IL-8水平与淋巴转移具有更强的相关性，在肺癌组织IL-8表达水平只与肿瘤的淋巴转移有关，与远处转移的相关性无显著的统计学意义。这一结果是IL-8的促血管形成作用所不能解释的，仔细分析病例资料，远处转移的患者（Ⅳ期）几乎均有较大范围的淋巴转移，所以本研究认为：IL-8、MMP-9的表达水平随肺癌进展而升高的总趋势主要是IL-8促淋巴生成作用的结果。

关于IL-8直接促进淋巴转移机制，目前未见文献报道。仅Krzystek-Korpacka等^[15]^报道食管鳞癌患者血清中IL-8和VEGF-C浓度具有明显的相关性，而VEGF-C具有促进淋巴管生成的作用，因此IL-8可能是通过促进淋巴管形成的作用来诱导淋巴转移的。因此进一步研究肺癌中IL-8和VEGF-C及淋巴转移的关系将是极有价值的工作。本研究还发现一个有趣的现象，即淋巴转移到N1患者血清中IL-8水平高于淋巴转移到N2患者的水平，因此我们猜测，IL-8在肿瘤转移的初期分泌较多，而在肿瘤转移后期，特别是有远处转移时，IL-8分泌已较少。

肿瘤的转移是一个主动过程，肿瘤细胞要脱离原发肿瘤组织，穿过细胞外基质和基底膜向淋巴管或血管中迁移，然后穿过血管基底膜，穿透血管腔，逃脱免疫监视，与远离原发灶器官的血管内皮细胞粘附，离开血管，在被侵入的微环境中存活和生长^[16]^。在此过程中，肿瘤细胞至少3次与基底膜相互作用，粘着并破坏基底膜^[2]^，因此细胞外基质（extracellular matrix, ECM）和基底膜是阻止肿瘤侵袭和转移的重要组织屏障。MMP是降解细胞外基质和基底膜最重要的酶类^[17]^。MMP-9属于MMP家族，它能降解破坏ECM中最主要的组分Ⅳ、Ⅴ型胶原和明胶^[6]^，已有文献报道在食管癌^[18]^、胃癌^[19]^、结直肠癌^[20, 21]^及乳腺癌^[22]^患者的肿瘤组织和血清中MMP-9表达水平明显升高。本研究显示，非小细胞肺癌患者MMP-9的血清水平和组织表达明显高于良性疾病对照组及正常对照组，这与Kopczynska等^[23]^报道类似。

近年来研究表明，IL-8可上调MMP的表达。Inoue等^[24]^研究认为IL-8通过上调MMP-9的表达，而参与肿瘤细胞的浸润和转移。Watanabe等^[25]^在进行口腔鳞状细胞癌试验时发现用重组IL-8处理细胞可使MMP-7 mRNA和蛋白的表达增高，而用抗IL-8抗体处理细胞时发现MMP-7 mRNA和蛋白的表达被抑制。

关于IL-8上调MMP表达的机制，许多学者进行了研究。Luca等^[26]^发现在黑色素瘤SB-2细胞系中，IL-8可在基因转录水平上调MMP-2 mRNA的表达，根据以前Sozzani等^[27]^发现在人中性粒细胞中，IL-8可激活磷脂酶D（phospholipase D, PLD），Reich等^[28]^发现在肿瘤细胞内，PLD介导的信号转导通路可诱导MMP-2的产生，他推测IL-8上调MMPs的可能机制为：细胞外的IL-8通过细胞膜表面受体激活细胞内的PLD介导的信号转导通路，PLD通路将信号传递给细胞核，刺激MMPs的转录及表达。而Subhadeep等^[29]^发现在中性粒细胞中细胞外的IL-8通过细胞膜表面受体激活细胞内的磷脂酶C（phospholipase C, PLC）介导的信号转导通路，PLC通路将信号传递给细胞核，刺激MMPs的转录及表达。本研究发现非小细胞肺癌患者组织和血清中IL-8、MMP-9表达均有明显的相关性，组织中的相关性最强（*r*=0.765），而在血清中二者的相关性相对减弱（*r*=0.445），这可能是由于在局部组织，受其它因素影响较小，IL-8上调MMP-9表达更易体现，而血清中的MMP-9不全是由于肿瘤细胞分泌释放入血的，单核细胞、间质细胞等细胞也可以分泌MMP-9，二者的相关性必然受到其它因素的影响。

根据以上的实验结果，我们分析了IL-8、MMP-9作为肿瘤标志物对非小细胞肺癌病情进展的评价效能，如表 4，可以看出，IL-8对于预测肺癌的淋巴转移具有较好的敏感性和特异性，但IL-8、MMP-9对于预测早期肺癌（尚未出现淋巴转移）的发生和远处转移的出现的效能较差。

综上所述，本研究结果表明：IL-8、MMP-9表达水平与非小细胞肺癌的进展密切相关，特别是IL-8与肺癌的淋巴转移具有明显的相关性，其机制可能是通过上调MMP-9实现的，但具体机制有待进一步研究。血清IL-8水平对于预测肺癌的淋巴转移具有较好的敏感性和特异性，可作为判断肺癌淋巴转移的参考。
